# An m6A-Related lncRNA Signature Predicts the Prognosis of Hepatocellular Carcinoma

**DOI:** 10.3389/fphar.2022.854851

**Published:** 2022-03-30

**Authors:** Zhenyu Zhang, Fangkai Wang, Jianlin Zhang, Wenjing Zhan, Gaosong Zhang, Chong Li, Tongyuan Zhang, Qianqian Yuan, Jia Chen, Manyu Guo, Honghai Xu, Feng Yu, Hengyi Wang, Xingyu Wang, Weihao Kong

**Affiliations:** ^1^ Anhui Key Laboratory of Bioactivity of Natural Products, School of Pharmacy, Anhui Medical University, Hefei, China; ^2^ Department of Emergency Surgery, Department of Emergency Medicine, The First Affiliated Hospital of Anhui Medical University, Hefei, China; ^3^ Department Ultrasound, The First Affiliated Hospital of Anhui Medical University, Hefei, China; ^4^ Department of Biochemistry and Molecular Biology, Metabolic Disease Research Center, School of Basic Medicine, Anhui Medical University, Hefei, China; ^5^ Department of Pathology, The First Affiliated Hospital of Anhui Medical University, Hefei, China; ^6^ Department of Emergency Medicine, The First Affiliated Hospital of Anhui Medical University, Hefei, China

**Keywords:** N6-methylandenosine, long non-coding RNA, prognosis, hepatocellular carcinoma, nomogram

## Abstract

**Objective:** The purpose of this study was to establish an N6-methylandenosine (m6A)-related long non-coding RNA (lncRNA) signature to predict the prognosis of hepatocellular carcinoma (HCC).

**Methods:** Pearson correlation analysis was used to identify m6A-related lncRNAs. We then performed univariate Cox regression analysis and least absolute shrinkage and selection operator (LASSO) Cox regression analysis to construct an m6A-related lncRNA signature. Based on the cutoff value of the risk score determined by the X-title software, we divided the HCC patients into high -and low-risk groups. A time-dependent ROC curve was used to evaluate the predictive value of the model. Finally, we constructed a nomogram based on the m6A-related lncRNA signature.

**Results:** ZEB1-AS1, MIR210HG, BACE1-AS, and SNHG3 were identified to comprise an m6A-related lncRNA signature. These four lncRNAs were upregulated in HCC tissues compared to normal tissues. The prognosis of patients with HCC in the low-risk group was significantly longer than that in the high-risk group. The M6A-related lncRNA signature was significantly associated with clinicopathological features and was established as a risk factor for the prognosis of patients with HCC. The nomogram based on the m6A-related lncRNA signature had a good distinguishing ability and consistency.

**Conclusion:** We identified an m6A-related lncRNA signature and constructed a nomogram model to evaluate the prognosis of patients with HCC.

## 1 Introduction

Hepatocellular carcinoma (HCC), the primary common type of liver cancer, ranks third among cancer-related deaths worldwide (Bray et al., 2018; Li et al., 2021). Despite advancements in current medical treatments, including liver resection (LR), transhepatic arterial chemotherapy and embolization (TACE), radiofrequency ablation (RFA), liver transplantation (LT), and targeted molecular therapy, the overall survival (OS) rate of liver cancer patients is still unsatisfactory (Omata et al., 2017). Traditional markers, including microvascular invasion, alpha-fetoprotein (AFP), tumor stage, and inflammation-related prognostic markers, have been used for a long time to predict the prognosis of patients with HCC (Kong et al., 2020c; Kong et al., 2021a; Kong et al., 2021b; Boilève et al., 2021). Still, the prognoses of patients with the same condition are sometimes very different. Therefore, it is of great clinical significance to identify easily detectable and accurate tumor prognostic markers. With the rapid development of bioinformatics technology in recent years, public databases, including TCGA (The Cancer Genome Atlas Program), ICGC (International Cancer Genome Consortium), and Gene Expression Omnibus (GEO), have collected a large amount of gene expression profiling data for mining, which provides a new strategy for developing tools for clinically predicting the prognosis of tumor patients (Kong et al., 2020a; Kong et al., 2020b; Zhang et al., 2021).

Since Desrosiers first described N6-methyladenosine (m6A) in 1974, researchers have discovered that it is a widely present modification of non-coding RNAs and mRNAs in eukaryotes (Fazi and Fatica, 2019; Dai et al., 2020; Yi Chen et al., 2020; Yi et al., 2020). Furthermore, numerous studies have indicated that m6A plays an essential role in carcinogenesis, including cervical cancer (CC), hepatocellular carcinoma (HCC), non-small cell lung cancer (NSCLC), thyroid cancer (TC), esophageal cancer (EC), gastric cancer (GC), breast cancer (BC), prostate cancer (PC), colorectal cancer (CRC), endometrial cancer (EC), and osteosarcoma (OS) (Zou et al., 2019; Anita et al., 2020; Hong Li et al., 2020; Guo et al., 2020; Kong et al., 2020a; Lin Zhang et al., 2020; Pan et al., 2020; Pei Wang et al., 2020; Shuo Chen et al., 2020; Tu et al., 2020; Zhao Ma et al., 2020). For example, Shuo Chen et al. (2020) found that erasers of ALKBH5 can inhibit the degradation of the oncogenic lncRNA PVT1, thereby promoting osteosarcoma progression. Long non-coding RNAs (lncRNAs) are RNA molecules whose transcript lengths exceed 200 nucleotides and do not encode proteins. Accumulating evidence indicates that abnormal lncRNA expression is related to the immune microenvironment, tumor occurrence, metabolism, invasion, migration, proliferation, and prognosis (Li et al., 2017; Bu et al., 2020; Ghafouri-Fard et al., 2020; Wang Li et al., 2020; Zhen-Jiang Ma et al., 2020). For example, Dong-Yan Zhang et al. (2020) found that UPK1A-AS1 is significantly increased in HCC tissues, which is significantly associated with the poor prognosis of patients with HCC, and promotes HCC cell line proliferation through interaction with EZH2. The main benefits of lncRNAs as prognostic cancer factors are their high sensitivity, specificity, stability, and non-invasiveness during fluid circulation. Owing to their cell/tissue specificity, it is easier to use lncRNAs for predicting the prognosis of cancer patients (Bolha et al., 2017). Considering m6A is one of the most common epigenetic modifications in mRNA and non-coding RNAs and plays an essential role in RNA stability, splicing, translation, and transportation (Li et al., 2022). To date, several studies have found that m6A can affect liver cancer progression by affecting lncRNA expression. Therefore, we chose m6A-related lncRNA to assess HCC patient prognosis, which provides potential targets for the treatment of HCC.

## 2 Materials and Methods

### 2.1 Dataset Source

We downloaded RNA-seq data (FPKM) and corresponding clinicopathological characteristics of HCC patients from the TCGA official website (https://portal.gdc.cancer.gov). ICGC RNA sequencing data and corresponding clinicopathological data were downloaded from the corresponding official website (https://dcc.icgc.org/projects/LIRI-JP). The HCC cohorts of TCGA and ICGC datasets contained RNA sequencing data of 370 and 232 HCC patients, respectively. Considering that HCC patients with a survival time of less than 30 days are likely to die due to non-tumor factors, we included 342 HCC patients in the TCGA cohort and 230 HCC patients in the ICGC cohort for subsequent analysis. According to previously published research of Tu et al., we identified 21 m6A regulatory factors, including readers (RBMX, HNRNPA2B1, HNRNPC, YTHDF3, YTHDF2, YTHDF1, IGF2BP3, IGF2BP2, IGF2BP1, YTHDC2, and YTHDC1), erasers (ALKBH5 and FTO), and writers (ZC3H13, RBM15B, RBM15, VIRMA, WTAP, METTL16, METTL14, and METTL3) (Tu et al., 2020). The expression matrix of the 21 m6A-regulators was then extracted from the TCGA and ICGC datasets for subsequent analysis.

### 2.2 Identification of m6A-Related lncRNAs in Hepatocellular Carcinoma

lncRNAs are defined as the following types: macro lncRNA, 3prime overlapping ncRNA, sense overlapping, sense intronic, processed transcript, antisense, and lincRNA (Tu et al., 2020; Yang et al., 2020). We downloaded the annotation file of lncRNAs from the GENCODE website (https://www.gencodegenes.org/) and annotated the ensemble IDs in the TCGA and ICGC datasets. Finally, we annotated 14,091 lncRNAs in the TCGA HCC dataset and 257 lncRNAs in the ICGC HCC dataset. The Perl language was used to extract the lncRNA expression matrix from TCGA and ICGC HCC datasets. Based on Wu’s research, we defined m6A-related lncRNAs as those with an absolute value of correlation coefficient between lncRNA expression and m6A regulators higher than 0.3, and a *p*-value less than 0.001 (Wu et al., 2020; Zhong et al., 2020; Jie Wu et al., 2021; Qianxue Wu et al., 2021).

### 2.3 Development and Validation of an m6A-Correlated lncRNA Prognostic Signature

We intersected the m6A-related lncRNAs determined in TCGA and ICGC datasets and obtained 24 common lncRNAs. In the TCGA training dataset, we used univariate Cox regression analysis to screen for m6A-related lncRNAs that were significantly related to the prognosis of patients with HCC. Then, m6A-related lncRNAs with a *p*-value of no more than 0.05 were incorporated into the Lasso Cox regression model to construct the predictive signature. The risk score was equal to the sum of the product of m6A-related prognostic lncRNA expression and its coefficient. X-tile software was used to determine the best cutoff value for TCGA HCC dataset risk score (Camp et al., 2004). We divided HCC patients into low -and high-risk groups using the TCGA dataset based on the selected cutoff value. The difference in survival outcomes of HCC patients in the high- and low-risk groups was analyzed using the log-rank test. The survivalROC package was used to draw a time-dependent ROC curve to evaluate the diagnostic value of m6A-related lncRNA risk scores in predicting the prognosis of patients with HCC. Based on the coefficients of the TCGA training dataset, we calculated the corresponding m6A-related lncRNA risk scores for patients with HCC patients in the ICGC dataset. Similarly, we determined the optimal cutoff value of the risk score of patients with HCC in the ICGC dataset based on X-title software. We divided the ICGC HCC cohort into high- and low-risk groups according to the cut-off value of the risk score in the ICGC dataset. Finally, we used the “SeqMap” software to re-annotate GSE14520-GPL3921 to obtain the expression matrix of lncRNAs, calculate the risk score using the previous method, divide the HCC patients in the GSE14520 cohort into a high- and a low-risk groups according to the optimal cutoff value, and explore the survival differences between high- and low-risk groups.

### 2.4 RNA Extraction and Quantitative Real-Time PCR

RNA was extracted from tissues using Trizol Reagent (Thermo Fisher, Cat. no. 15596026, United States) according to the manufacturer’s instructions. The PCR-specific primers were determined using GENERAL BIOL (Anhui, China) and used according to the manufacturer’s instructions. RNA was converted to cDNA using the PrimeScript RT Polymerase (Takara) reverse transcription kit to determine lncRNA mRNA expression. The fold change was calculated using the 2^−ΔΔCT^ method, with β-actin as an internal control. The primer sequences were as follows: ZEB1-AS forward 5-CCG​TGG​GCA​CTG​CTG​AAT-3 and reverse 5-CTG​CTG​GCA​AGC​GGA​ACT-3; MIR210HG forward 5- GCA​GGC​ACA​GGT​GTG​GTC​ATA​TC-3 and reverse 5′-AGGCAGGCTCAGCA GACAGG-3′; BACE1-AS forward 5- GAA​GGG​TCT​AAG​TGC​AGA​CAT​CTT-3 and reverse 5-AGG​GAG​GCG​GTG​AGA​GT-3; SNHG3 forward 5-TTCAAG CGATTCTCGTGCC-3 and reverse 5-AAG​ATT​GTC​AAA​CCC​TCC​CTG​T-3; and β-actin forward 5-CCC​ATC​TAT​GAG​GGT​TAC​GC-3 and reverse 5-TTT​AAT​GTC​ACG​CAC​GAT​TTC-3′.

### 2.5 Nomogram Construction

To explore whether m6A-related lncRNA risk score is an independent factor predicting the prognosis of patients with HCC, we also included clinical data such as age, sex, TNM stage, and histological grade into the Cox regression model. Univariate and multivariate Cox regression analyses were performed sequentially to screen for independent risk factors that affected the prognosis of patients with HCC. We constructed a nomogram based on variables selected using multivariate Cox regression analysis. The nomogram’s distinguishing ability was determined using the C-index and the area under the time-dependent ROC curve, and its consistency is shown using the 1-year, 2-year, and 3-year calibration curves.

### 2.6 Gene Set Enrichment Analysis

The Gene set enrichment analysis (GSEA) (http://software.broadinstitute.org/Gsea/downloads.jsp) was used to explore the potential pathways for the role of m6A-related lncRNAs in HCC. Based on the selected cutoff value for the TCGA dataset, the TCGA HCC cohort was divided into low- and high-risk groups. Therefore, an annotation file (.cls) required by GSEA was obtained. Next, we imported the expression profile containing 56,753 genes and 342 HCC samples into the Morpheus website (https://software.broadinstitute.org/morpheus/) to obtain the expression matrix (.gct), as determined by the GSEA. Finally, we uploaded these two files to GSEA software and set the gene set to c2.cp.kegg.v7.2.symbols.gmt. The permutation parameter was set to 1,000. A *p*-value less than 0.05 and FDR less than 0.25 were considered to indicate statistically significant differences.

### 2.7 Correlation Between m6A-Related lncRNA Signature and Immune Infiltration

The timer database (https://cistrome.shinyapps.io/timer/) contains six immune cell components in the TCGA dataset, including CD8 T cells, neutrophils, CD4 T cells, B cells, macrophages, and dendritic cells. Based on the value of the m6A-related lncRNA risk score constructed in the TCGA dataset, we performed Spearman’s correlation analysis to explore the correlation between the risk score and the level of immune cell infiltration.

### 2.8 Statistical Analyses

We used different statistical description methods based on different types of variables. We used the mean ± standard deviation (SD) to describe the variables that met the normal distribution and used the median (interquartile range) (IQR) to describe the variables that did not meet the normal distribution. Pearson correlation analysis was used to analyze the correlation between m6A regulators and lncRNAs in TCGA and ICGC HCC datasets. Statistical analysis was performed using R software (version 4.0.3.) and the SPSS software (version 25.0). A double-sided *p*-value of less than 0.05 is defined as statistically significant.

## 3 Results

### 3.1 Patient Characteristics and Processing

**FIGURE 1:**
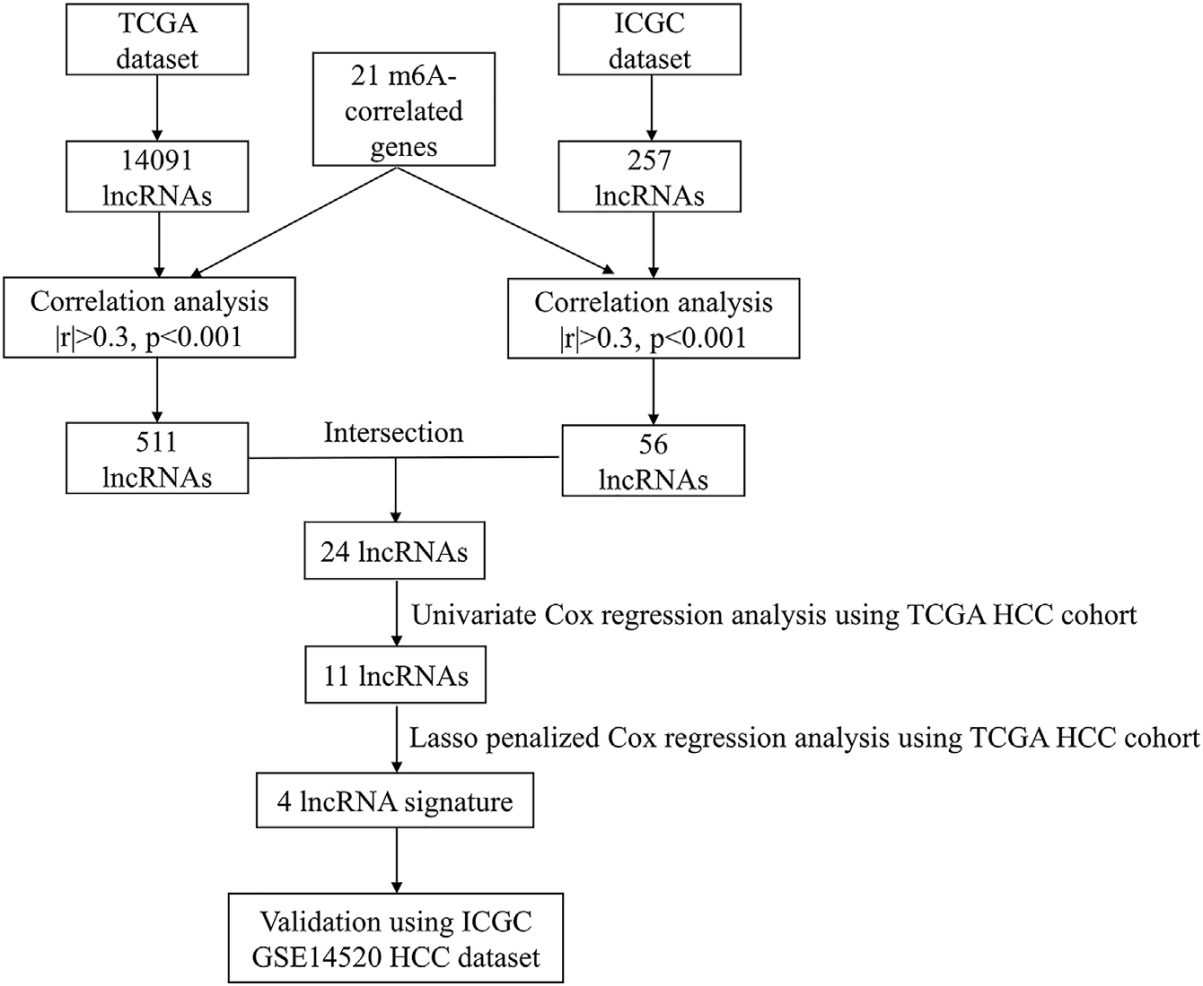
Flowchart of the research. HCC, Hepatocellular carcinoma; TCGA, The Cancer Genome Atlas; ICGC, International Cancer Genome Consortium.

After excluding patients with HCC with missing survival time or survival time of less than 30 days, we finally included 342 HCC patients in the TCGA cohort and 230 HCC patients in the ICGC cohort for follow-up analysis. Detailed information on the patients with HCC included in this study is presented in Table 1. Figure 1 presents a detailed flowchart of our research. TCGA dataset was used to construct the m6A-related lncRNA signature, and the ICGC and GSE14520 datasets were used to verify the established predictive signature.

**TABLE 1:** Baseline characteristics of HCC patients included in this study.

Variables	TCGA	ICGC
	*n* = 342	*n* = 230
Age, years	61.0 (18.0)	67.3 (12.0)
Sex, male/female	233/109	169/61
Histological grade, G1/G2/G3/G4/null	53/161/111/12/5	—
TNM Stage, I/II/III/IV/null	161/77/80/3/21	36/106/69/19
Prior-Maligancy, no/yes	—	200/30
Survival status, Alive/Dead	219/123	189/41

HCC, Hepatocellular carcinoma; TNM, Tumor Node Metastasis; TCGA, The Cancer Genome Atlas; ICGC, International Cancer Genome Consortium.

### 3.2 Construction of an m6A-Related lncRNA Prognostic Signature

**FIGURE 2:**
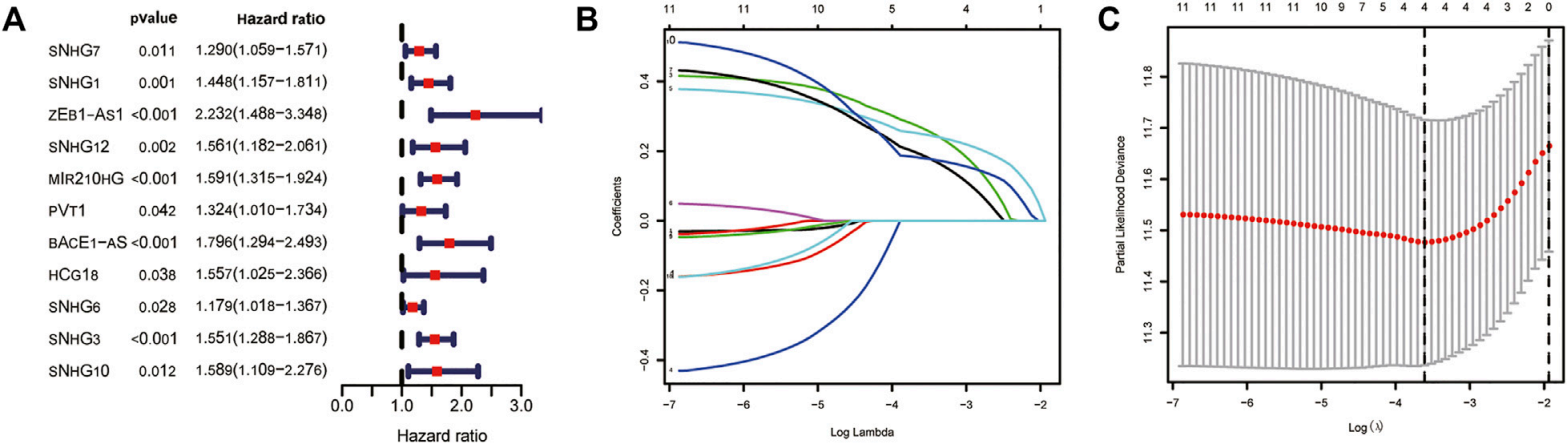
Construction of m6A-related lncRNA signature using the TCGA dataset. **(A)** The univariate Cox regression model identifies lncRNAs that affect overall survival; **(B,C)** Lasso Cox regression model to identify the best penalty parameter. TCGA, The Cancer Genome Atlas; Lasso, Least absolute shrinkage and selection operator.

**FIGURE 3:**
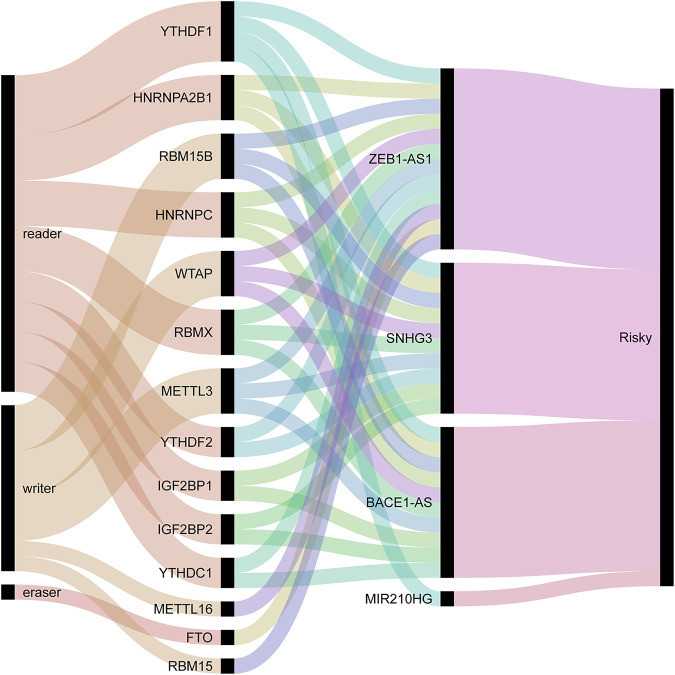
Sankey diagram between m6A regulators and the selected four lncRNAs.

**FIGURE 4:**
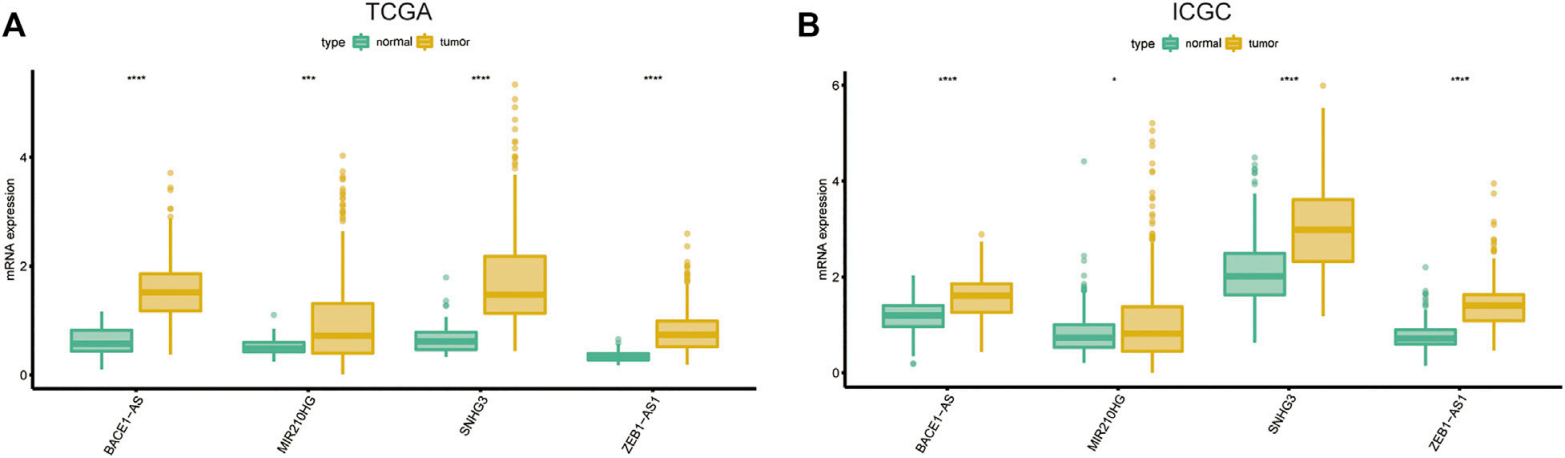
The expression of the four screened lncRNAs in HCC tissues and normal tissues in the TCGA **(A)** and ICGC **(B)** dataset. HCC, Hepatocellular carcinoma; TCGA, The Cancer Genome Atlas; ICGC, International Cancer Genome Consortium.

**FIGURE 5:**
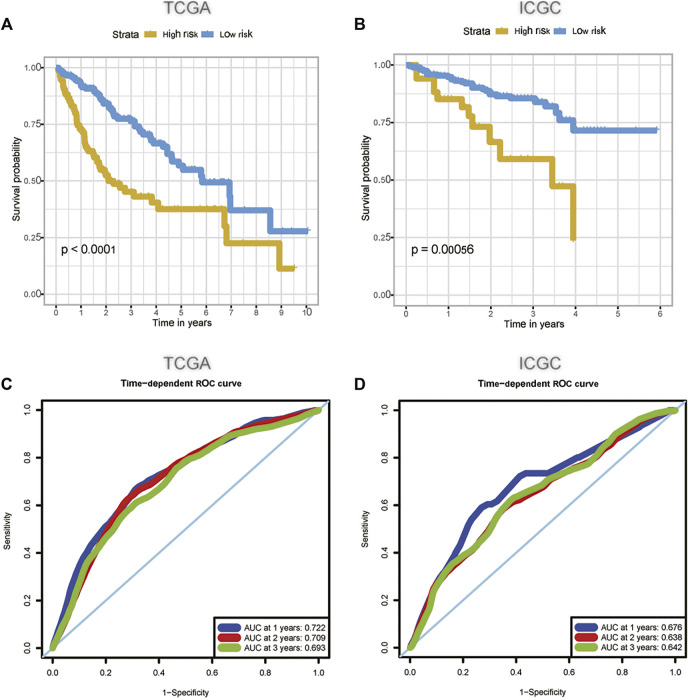
The predictive value of m6A-related lncRNA signature in the TCGA **(A, C)** and the ICGC **(B, D)** dataset. **(A)** The survival curve of HCC patients in the high and low-risk groups in the TCGA dataset; **(B)** The survival curve of HCC patients in the high and low-risk groups in the ICGC dataset; **(C)** The ability of m6A-related lncRNA signature to predict the overall survival of HCC patients in the TCGA dataset; **(D)** The ability of m6A-related lncRNA signature to predict the overall survival of HCC patients in the ICGC dataset. HCC, hepatocellular carcinoma; TCGA, The Cancer Genome Atlas; ICGC, International Cancer Genome Consortium.

**FIGURE 6:**
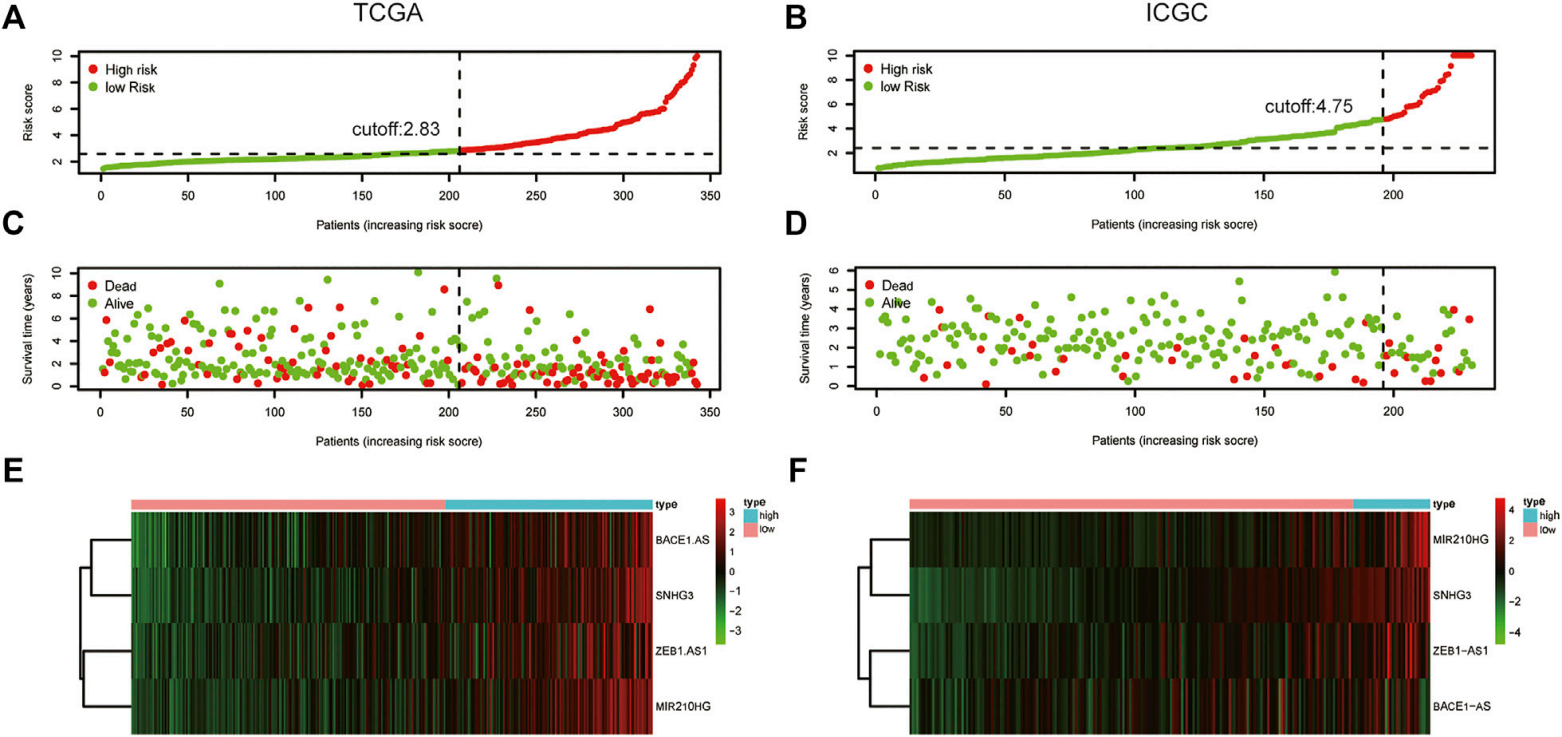
The distribution of risk score, survival status, and four lncRNAs expression. **(A)** The distribution of m6A-related lncRNA risk score in the TCGA dataset; **(B)** The distribution of m6A-related lncRNA risk score in the ICGC dataset; **(C)** The distribution of the survival status of HCC patients in the TCGA dataset; **(D)** The distribution of the survival status of HCC patients in the ICGC dataset; **(E)** The expression matrix heatmap of the selected four m6A-related lncRNAs in the TCGA dataset; **(F)** The expression matrix heatmap of the selected four m6A-related lncRNAs in the ICGC dataset. HCC, Hepatocellular carcinoma; TCGA, The Cancer Genome Atlas; ICGC, International Cancer Genome Consortium.

We used univariate Cox regression analysis to explore the relationship between m6A-related lncRNAs and overall survival of HCC patients in the TCGA cohort. The results of the univariate Cox regression model showed that 11 m6A-related lncRNAs were significantly associated with the overall survival of patients with HCC (*p* <0.05) (Figure 2A). We then included these 11 lncRNAs in the Lasso Cox regression model and obtained the best penalty parameters through ten-fold cross-validation based on the glmnet package (Figures 2B,C). Finally, we identified four m6A-LncRNAs to predict the prognosis of patients with HCC (Table 2). These four m6A-related lncRNAs were risk factors for the prognosis of patients (Figure 3). The heatmap of the correlations between the four screened m6A-related lncRNAs and all m6A regulators is shown in Supplementary Figure S1. Moreover, compared with the expression levels in normal tissues, the expression of these four lncRNAs was significantly increased in tumor tissues, which is consistent with our expected results (Figures 4A,B). According to the results of quantitative real-time PCR, we found that the expression of lncRNA ZEB1-AS1, lncRNA BACE1-AS, lncRNA MIR210HG, and lncRNA SNHG3 in multiple liver cancer tissue samples and their corresponding adjacent tissues was significantly increased in liver cancer (Supplementary Figure S2). Therefore, we constructed an m6A-related lncRNA risk score. The risk score formula was as follows: risk score = 0.2679*ZEB1−AS1+0.2496*MIR210HG+0.1902*BACE1−AS+0.1807* SNHG3. Based on the selected cutoff value (2.83) of the risk score in the TCGA cohort determined by the X-title software, we divided the TCGA HCC cohort into low- and high-risk groups. Compared to the high-risk group, patients in the low-risk group had a longer overall survival time (*p* <0.0001) (Figure 5A). Moreover, the area under the ROC curve for 1-, 2, and 3 years-OS was 0.722, 0.709, and 0.693, respectively (Figure 5C). Hence, the m6A-related lncRNA risk score has moderate diagnostic value for predicting the overall survival of HCC patients in the TCGA dataset. The distribution of m6A-related lncRNA risk scores in the TCGA dataset is shown in Figure 6A. Figure 6C shows the distribution of survival status of patients with HCC in the TCGA dataset. Figure 6E shows the expression matrix heatmap of the four selected m6A-related lncRNAs in the TCGA dataset.

**TABLE 2:** Four selected m6A-correlated prognostic lncRNAs.

M6A-Correlated lncRNA	Biotype	Coefficient of lasso regression	HR	HR.95L	HR.95H	*p*-Value
ZEB1-AS1	antisense	0.2679	1.5609	1.1821	2.0612	**0.0017**
MIR210HG	lincRNA	0.2496	1.5909	1.3155	1.9241	**0.0000**
BACE1-AS	processed_transcript	0.1902	1.7961	1.2941	2.4928	**0.0005**
SNHG3	processed_transcript	0.1807	1.5512	1.2885	1.8674	**0.0000**

HR, Hazard ratio.

Bold fonts represent P-values less than 0.05.

### 3.3 Validation of the Predictive Value of the m6A-Related lncRNA Prognostic Signature

According to our previously constructed m6A-related lncRNA risk score formula, we calculated the corresponding risk scores for patients with HCC in the ICGC dataset. Based on the X-title software, we determined the cutoff value of the m6A-related lncRNA risk score in the ICGC dataset to 4.75 and then divided the HCC patients into low- and high-risk groups. The survival curve analysis results indicated that HCC patients in the low-risk group had significantly longer overall survival compared with HCC patients in the high-risk group (Figure 5B). The areas under the ROC curve for 1-, 2, and 3 years-OS were 0.676, 0.638, and 0.642, respectively. Hence, the m6A-related lncRNA risk score also had a moderate diagnostic value for predicting the overall survival of patients with HCC in the ICGC dataset (Figure 5D). The distribution of m6A-related lncRNA risk scores in the ICGC dataset is shown in Figure 6B. Figure 6D shows the distribution of the survival status of patients with HCC in the ICGC dataset. Figure 6F shows the expression matrix heatmap of the four selected m6A-related lncRNAs in the ICGC dataset. Finally, in the GSE14520 dataset, “SeqMap” software re-annotated the platform of GPL3921, and only three annotated lncRNAs (SNHG3 BACE1-AS ZEB1-AS) were annotated. We emulated the method used by Tang et al., (Tang et al., 2021), so our formula for calculating the m6A-related lncRNA risk score is: risk score = 0.1807*SNHG3+ 0.1902*BACE1−AS + 0.2679*ZEB1-AS. According to the optimal cutoff value (6.96132), we divided the patients with HCC into high- and low-risk groups. The overall survival time of patients with liver cancer in the high-risk group was lower than that in the low-risk group (*p* = 0.017) (Supplementary Figure S3A). The results of time-dependent ROC curve analysis showed that the m6A-related lncRNA risk scores predicted the 1-year, 2-year, and 3-year areas under the ROC curve of patients with HCC, which were 0.574, 0.573, and 0.591, respectively (Supplementary Figure S3B). This poor predictive value may be attributed to the lack of MIG210HG expression in the GSE14520-GPL3921 dataset. Therefore, a larger cohort of samples is required for external validation.

### 3.4 Correlation of the m6A-Related lncRNA Signature With Clinicopathological Features

Correlation analysis was employed to explore the association between m6A-related lncRNA signatures and clinicopathological features of HCC patients. In the TCGA dataset, 319 patients with full clinicopathological information (including age, sex, histologic grade, TNM stage, and survival status) were analyzed. The m6A-related lncRNA signature was significantly associated with sex (*p* = 0.023), histological grade (*p* <0.001), TNM stage (*p* = 0.002), and survival status (*p* <0.001). In the ICGC dataset, we found that the m6A-related lncRNA signature was significantly associated with sex (*p* = 0.012), TNM stage (*p* = 0.022), and survival status (*p* = 0.004), which is consistent with the results of TCGA (Table 3). In the GSE14520 dataset, we found that the m6A-related lncRNA signature was significantly related to the TNM stage (*p* = 0.004) (Supplementary Table S1).

**TABLE 3:** Association between m6A-correlated lncRNA signature and clinicopathologic characteristics in HCC cohort.

Variables	TCGA (*n* = 319)		ICGC (*n* = 230)			
	Low risk group	High risk group	*p*-Value	Low risk group	High risk group	*p*-Value
Age (years)			0.766			0.528
≤60	95	65		37	8	
>60	97	62		159	26	
Sex			**0.023**			**0.012**
Male	141	78		150	19	
Female	51	49		46	15	
Prior-malignancy						0.810
No	—	—		170	30	
Yes	—	—		26	4	
Histologic grade			**<0.001**			
G1/2	137	61		—	—	
G3/4	55	66		—	—	
TNM stage			**0.002**			**0.022**
I/II	154	82		127	15	
III/IV	38	45		69	19	
Survival status			**<0.001**			**0.004**
Alive	146	66		167	22	
Dead	46	61		29	12	

HCC, Hepatocellular carcinoma; TNM, Tumor Node Metastasis; TCGA, The Cancer Genome Atlas; ICGC, International Cancer Genome Consortium.

Bold fonts represent P-values less than 0.05.

### 3.5 The m6A-Related lncRNA Signature is Associated With the Prognosis of Patients With Hepatocellular Carcinoma

Univariate and multivariate Cox regression models were used to determine the independent risk variables for the prognosis of patients with HCC. In the TCGA dataset, we included age, sex, TNM stage, histologic grade, and risk score in the univariate Cox regression model. Univariate Cox regression analysis showed that TNM stage (*p* <0.001) and risk score (*p* <0.001) were significantly related to the overall survival of patients with HCC. We then input these two variables into the multivariate Cox regression analysis, and the results showed that the m6A-related lncRNA risk score was an independent risk factor for the overall survival of patients with HCC (*p* <0.001) (Table 4). Similarly, the ICGC dataset also confirmed that the m6A-related lncRNA risk score was an independent risk factor for the overall survival of patients with HCC (*p* = 0.025) (Table 5). However, in the GSE14520 dataset, the results of the univariate Cox regression analysis indicated that TNM stage (*p* <0.001), cirrhosis (*p* = 0.032), and risk score (*p* = 0.022) were significantly associated with OS. Multivariate Cox regression analysis showed that TNM stage (*p* <0.001) was an independent risk factor for OS in patients with HCC (Supplementary Table S2).

**TABLE 4:** Univariate and multivariate regression analyses of OS in HCC patients of the TCGA dataset.

Variable	Univariate analysis			Multivariate analysis		
	*P*	Hazard ratio	95% Confidence interval	*p*	Hazard ratio	95% Confidence Interval
Age (>60/≤60)	0.414	1.172	0.800–1.717			
Sex (female/male)	0.162	1.320	0.894–1.948			
TNM stage (IV + III/II + I)	**<0.001**	**2.835**	**1.934–4.157**	**<0.001**	**2.614**	**1.778–3.841**
Histologic grade (G4+G3/G2+G1)	0.660	1.091	0.739–1.611			
Risk score (high/low)	**<0.001**	**2.713**	**1.846–3.987**	**<0.001**	**2.522**	**1.710–3.719**

TNM, Tumor Node Metastasis; OS, overall survival; HCC, Hepatocellular carcinoma; TCGA, The Cancer Genome Atlas.

Bold fonts represent P-values less than 0.05.

**TABLE 5:** Univariate and multivariate regression analyses of OS in HCC patients of the ICGC dataset.

Variable	Univariate analysis			Multivariate Analysis		
	*p*	Hazard Ratio	95% Confidence interval	*p*	Hazard ratio	95% Confidence interval
Age (>60/≤60)	0.483	0.774	0.379–1.581			
Sex (female/male)	**0.022**	**2.098**	**1.115–3.948**	**0.019**	**2.242**	**1.144–4.392**
TNM stage (IV + III/II + I)	**0.012**	**2.203**	**1.191–4.076**	**0.009**	**2.357**	**1.236–4.495**
Prior-Malignancy (yes/no)	0.131	1.885	0.829–4.289			
Risk score (high/low)	**0.001**	**3.089**	**1.570–6.078**	**0.025**	**2.238**	**1.106–4.531**

TNM, Tumor Node Metastasis; OS, overall survival; HCC, Hepatocellular carcinoma; ICGC, International Cancer Genome Consortium.

Bold fonts represent P-values less than 0.05.

### 3.6 Building and Validation of Nomogram Based on the m6A-lncRNA Signature

**FIGURE 7:**
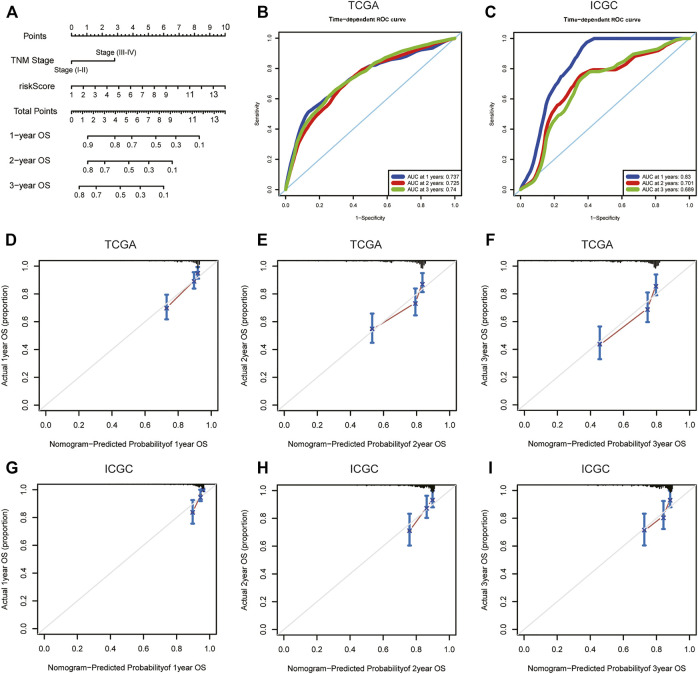
OS-related nomogram construction. **(A)** The intuitive nomogram model is used to predict the 1-year, 2-year, and 3-year OS rates of HCC patients; **(B)** The predictive value of the nomogram for the 1-year, 2-year, and 3-year OS rates of HCC patients in TCGA dataset; **(C)** The predictive value of the nomogram for the 1-year, 2-year, and 3-year OS rates of HCC patients in external ICGC dataset; **(D–F)** 1-year, 2-year, and 3-year calibration curve of OS-related nomogram in the TCGA dataset; **(G–I)** 1-year, 2-year, and 3-year calibration curve of OS-related nomogram in the ICGC dataset. OS, overall survival; TCGA, The Cancer Genome Atlas; ICGC, International Cancer Genome Consortium.

**FIGURE 8:**
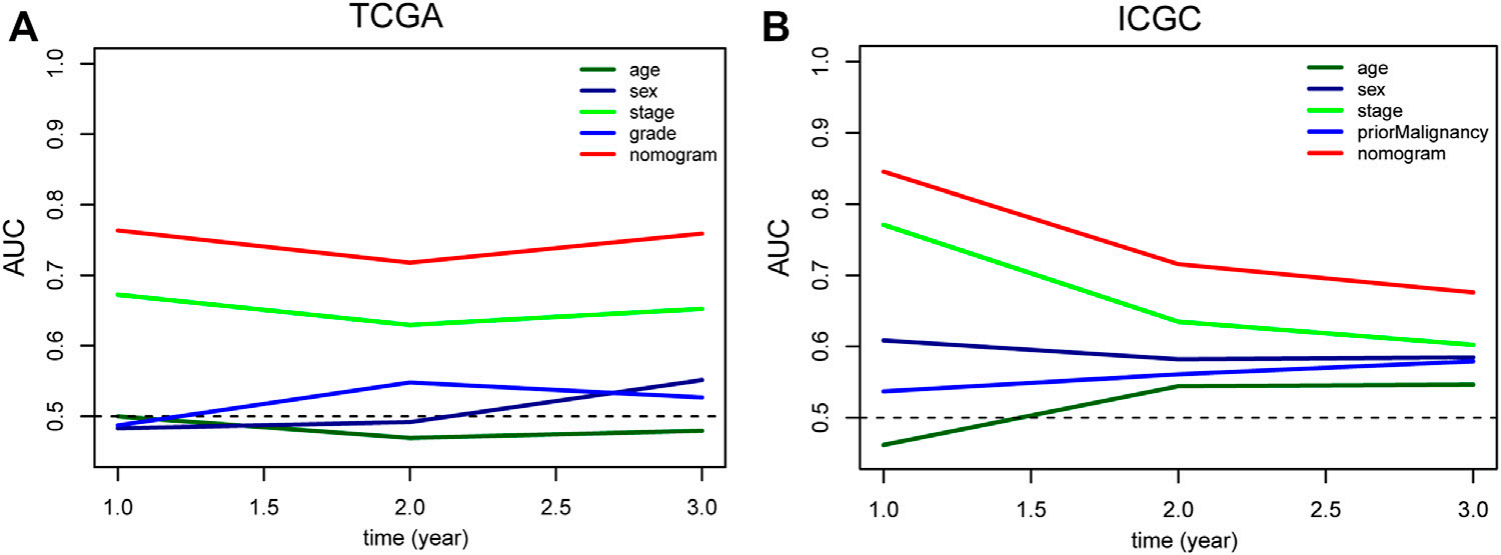
Comparison of the predictive value of nomogram and other clinicopathological features in TCGA **(A)** and ICGC **(B)** HCC cohort. TCGA, The Cancer Genome Atlas; ICGC, International Cancer Genome Consortium; HCC, Hepatocellular carcinoma.

To intuitively assess the prognosis of patients with HCC, we established a predictive nomogram model. According to the multivariate Cox regression analysis results, we included the m6A-related lncRNA risk scores and TNM stages to construct a nomogram model (Figure 7A). In the TCGA dataset, the OS-related nomograms we created had a better predictive ability for the 1-year, 2-year, and 3-year overall survival rates of patients with HCC. The area under the 1-year, 2-year, and 3-year ROC curves for the OS-related nomograms were 0.737, 0.725, and 0.740, respectively (Figure 7B). The calibration curve results showed that the nomogram we constructed predicts the 1-year, 2-year, and 3-year survival rates of HCC patients with good consistency with the actual survival rates (Figures 7D–F). At the same time, we used the ICGC dataset to verify the accuracy of the OS nomogram we constructed, and the results showed that the nomogram in the ICGC dataset has good predictive value for evaluating the 1-, 2, and 3 year-overall survive. The area under the 1-year, 2-year, and 3-year ROC curves for OS-related nomograms were 0.830, 0.701, and 0.689, respectively (Figure 7C). The calibration curve results also showed good consistency between the actual and predicted OS (Figures 7G–I). In the GSE14520 dataset, the area under the 1-year, 2-year, and 3-year ROC curves for the OS-related nomograms were 0.668, 0.712, and 0.689, respectively (Supplementary Figure S4A). The calibration curve results showed that the nomogram predicted the 1-year, 2-year, and 3-year survival rates of HCC patients with good consistency with the actual survival rates (Supplementary Figures S4C–E). Finally, we compared the constructed nomogram model with other clinicopathological features to predict the prognosis of patients with HCC. The results showed that, when compared with other clinicopathological features, the nomogram model had better diagnostic value in the TCGA training cohort and the ICGC/GSE14520 validation cohort (Figure 8; Supplementary Figure S4B).

### 3.7 Gene Set Enrichment Analysis

**FIGURE 9:**
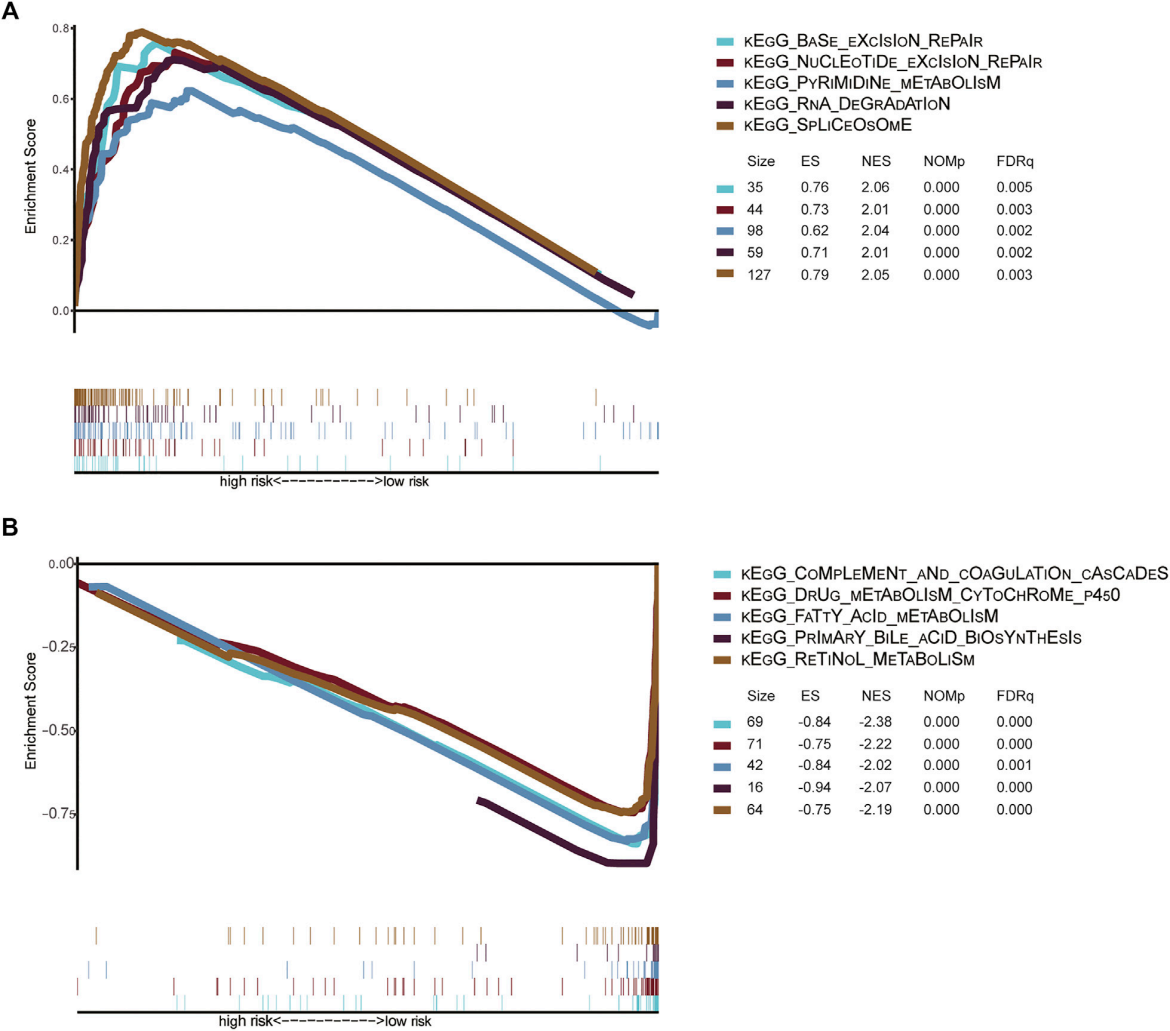
**(A)** Signaling pathways enriched in high-risk groups identified by GSEA **(B)** Signaling pathways enriched in low-risk groups identified by GSEA Gene set enrichment analysis of high-risk and low-risk groups in the TCGA HCC dataset. KEGG, Kyoto Encyclopedia of Genes and Genomes; TCGA, The Cancer Genome Atlas; GSEA, gene set enrichment analysis; ES, Enrichment score; NES, Normalized enrichment score.

To further identify the molecular mechanism of the m6A-related lncRNA signature in patients with HCC, we divided 342 HCC patients in the TCGA dataset into high- and low-risk groups based on the previous cutoff value (2.83). The enrichment pathways of the high-risk group were mainly enriched in complement and coagulation cascades, drug metabolism cytochrome P450, retinol metabolism, primary bile acid biosynthesis, and fatty acid metabolism (Top 5). The enrichment pathways of the high-risk group were mainly concentrated in base excision repair, spliceosome, pyrimidine metabolism, RNA degradation, and nucleotide excision repair (Top 5) (Figure 9). The detailed results of the GSEA of the high- and low-risk groups are shown in Supplementary Tables S3, S4.

### 3.8 The m6A-Related lncRNA Signature Correlated With Immune Infiltration

**FIGURE 10:**
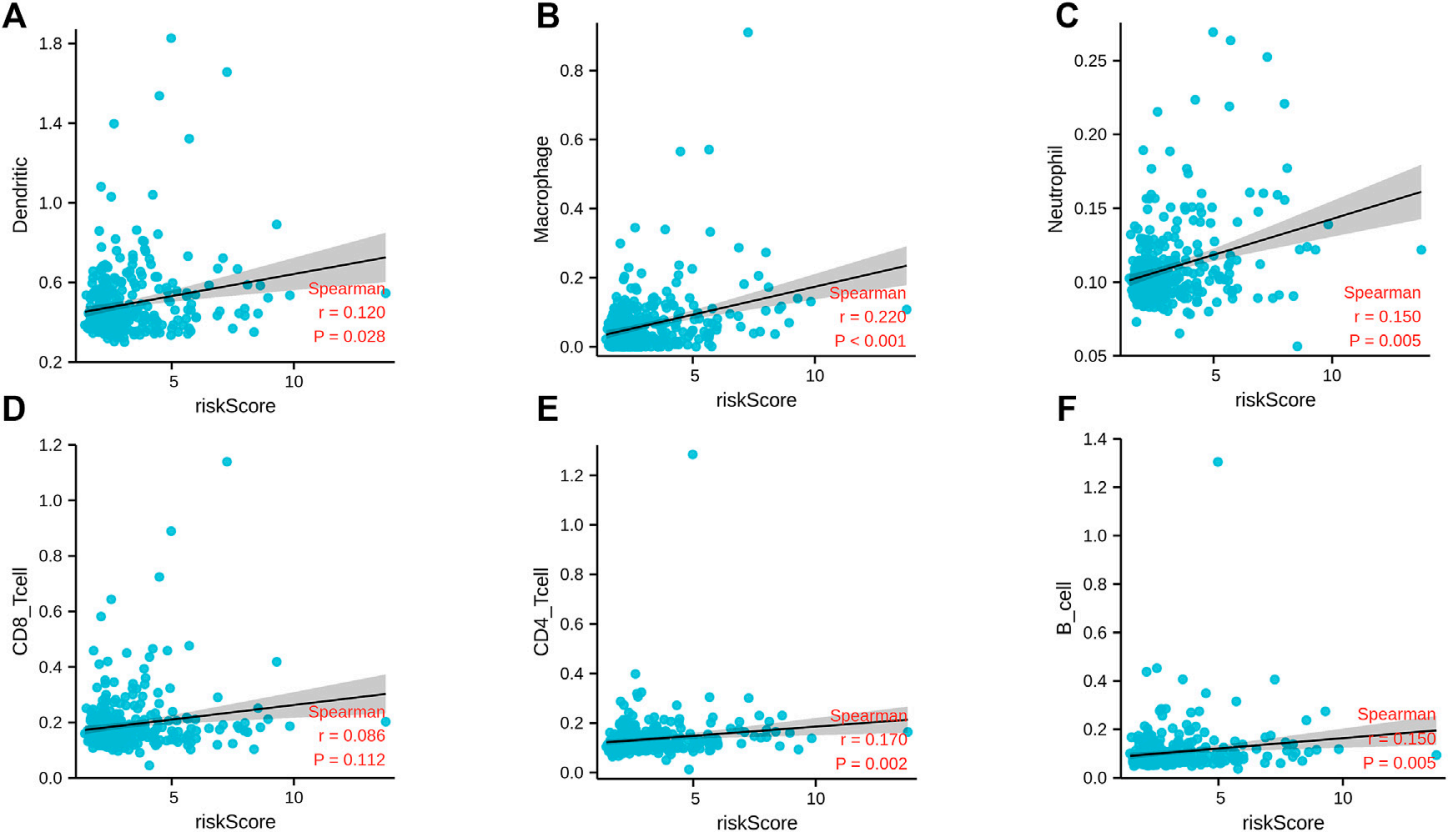
Correlation analysis between m6A-correlated lncRNA risk score and immune cell infiltration. **(A)** Dendritic; **(B)** macrophage; **(C)** neutrophils; **(D)** CD8 T cells; **(E)** CD4 T cells; **(F)** B cells.

At the same time, we also explored the correlation between the m6A-related lncRNA risk score and the immune infiltration. The results of correlation analysis showed that the m6A-related lncRNA risk score was significantly related to neutrophils (*r* = 0.150; *p* = 0.005), CD4 T cells (*r* = 0.170; *p* = 0.002), B cells (*r* = 0.150; *p* = 0.005), macrophages (*r* < 0.220; *p* < 0.001), and dendritic cells (*r* = 0.120; *p* = 0.028) (Figure10). This shows that the immune microenvironment may affect the prognosis of patients with liver cancer.

## 4 Discussion

With the deepening of lncRNAs research in recent years, their correlation with m6A regulators has gradually attracted the attention of scientific researchers. There has been a lot of published literature about m6A regulators or lncRNA signature prediction models used to predict the prognosis of patients with tumors, especially HCC (Kong et al., 2020a; Wang Li et al., 2020). For example, Kong et al. found that the m6A regulator risk score constructed using YTHDF1 and YTHDF2 can predict the prognosis of patients with HCC and is also an independent risk factor for their prognosis (Kong et al., 2020a). Through the analysis of the TCGA dataset, Li et al. constructed an eleven-lncRNA predictive signature of HCC, which can significantly distinguish high-risk patients among patients with HCC and better predict their prognosis (Wang Li et al., 2020). However, to date, no relevant research has systematically explored an HCC prediction model of m6A-related lncRNAs to assess prognosis of patients with HCC. Therefore, in this study, we constructed an m6A-related lncRNA signature of HCC to provide new ideas for predicting the prognosis of HCC patients.

OPLS-DA is a supervised statistical discriminant analysis (LDA) method. This method uses partial least squares regression to establish a relationship between lncRNA expression and dependent variables. It uses orthogonal signal correction technology to decompose the X matrix information into two types of information related to Y and unrelated to Y, and then filters out the information irrelevant to the classification. The relevant information is mainly concentrated in the first prediction component. It can effectively reduce the complexity of the model and enhance its explanatory power without reducing its predictive ability. It is generally believed that in binary classification questions, OPLS-DA can be used as the preferred analysis method, which is often used in metabolomic studies (Szymańska et al., 2012; Cao et al., 2017). Robert Tibshirani first proposed the LASSO model in 1996. It produces a more refined model by constructing a penalty function to compress some regression coefficients, forcing the sum of the absolute values of the coefficients to be less than a fixed value; at the same time, some regression coefficients are set as zero. Therefore, the advantage of subset shrinkage is preserved, which is a biased estimation when dealing with complex collinear data (Zhiwei Wu et al., 2021). In essence, both OPLS-DA and LASSO are linear regression models, but OPLS-DA adopts the idea of component decomposition, whereas LASSO adopts a penalty constraint strategy. OPLS-DA focuses on the Y interpretation of each variable and retains the collinear variables. The principle of LASSO is to make the model as simple as possible and to eliminate collinear variables.

In our study, through the integration of TCGA and ICGC datasets, we determined an m6A-related lncRNA signature for predicting the prognosis of patients with HCC. Based on the cutoff value of the m6A-related lncRNA risk score for each dataset, we divided the patients with HCC into low-and high-risk groups. The overall survival rate of patients with HCC in the high-risk group was significantly lower than that in the low-risk group. In the TCGA training dataset, the results of multivariate Cox regression analysis showed that the risk score and tumor stage were independent risk factors for the prognosis of patients with HCC; therefore, we constructed a nomogram model based on these two indicators. The nomogram had a moderate ability to predict prognosis of patients with HCC and could also accurately predict the prognosis of these patients in the external validation dataset. Using the nomogram model we constructed, we predicted the 1-year, 2-year, and 3-year survival rates of each patient with HCC based on the expression of these four lncRNAs in the tumor tissue of each patient and the tumor stage. The higher the overall score, the worse the 1-year, 2-year, and 3-year survival rates of patients with HCC. This is based only on the results of the public database, and multi-center data are needed for verification in the future. Suppose the reliability of the model is verified in a multi-center cohort, the corresponding lncRNA panel could be developed for clinical applications to provide medical advice. It can be used to predict the prognosis of patients with liver cancer.

ZEB1-AS1 is transcribed by the bidirectional promoter of ZEB1. In our study, ZEB1-AS1 was significantly increased in liver cancer tissues and significantly associated with poor prognosis in patients with liver cancer. Li et al.’s (2016)s research found that ZEB1-AS1 promotes tumor growth and metastasis and is significantly associated with tumor recurrence. Zhen-Jiang Ma et al. (2020) found that ZEB1-AS1 can promote bone metastasis in liver cancer through the miR-302b-EGFR-PI3K-AKT axis. These results are consistent with our research.

MIR210HG is transcribed from ENSG00000247095.2 present on human chromosome 11p15.5. MIR210HG is elevated in a variety of tumors such as cervical cancer, non-small cell lung cancer, breast cancer, colorectal adenocarcinoma, pancreatic cancer, liver cancer, osteosarcoma, and glioma (Min et al., 2016; Li et al., 2017; Li et al., 2019; Wang et al., 2019; Ai-Hong Wang et al., 2020). For example, Wang et al.’s (2019) research found that increased expression of MIR210HG was significantly related to the malignant progression of tumors, and *in vitro* studies further confirmed that silencing MIR210HG significantly reduced the proliferation, invasion, and migration capabilities of liver cancer cell lines. These results are consistent with those of our research.

Since BACE1-AS was found to be abnormally expressed in Alzheimer’s disease by Faghihi et al. (2008), it has been shown to play an essential role in cancer, including gastric and ovarian cancers (Chen et al., 2016; Esfandi et al., 2019). At present, no relevant studies have explored its molecular mechanisms in liver cancer. Hence, further experiments are required to explore the mechanism of BACE1-AS in HCC.

SNHG3 plays a vital role in the occurrence and development of many tumors, including liver cancer (Wu et al., 2019; Zhang et al., 2019). In our study, the expression of SNHG3 in liver cancer tissues was significantly increased, and it was significantly associated with poor prognosis in patients with liver cancer. Wu et al.’s (2019) research found that SNHG3 can target miR-139-5p to regulate the expression of BMI1, thereby promoting the migration, invasion, and proliferation of liver cancer cells. Zhang et al.’s (2019) study found that SNHG3 induces epithelial-mesenchymal transition and resistance to sorafenib by regulating the miR-128/CD151 pathway in HCC. This finding is consistent with the results of our study.

Although our study obtained a novel m6A-related lncRNA signature for predicting prognosis of patients with HCC, it also has some limitations. First, the interactions between m6A regulatory factors and lncRNAs should be experimentally verified. Second, because of the limited number of HCC datasets with prognostic information and lncRNA gene symbols, we used only the ICGC dataset for external verification.

In conclusion, we constructed an m6A-related lncRNA signature to predict the prognosis of HCC patients and also constructed a predictive nomogram that can more accurately predict the prognosis of HCC patients and provide guidance for individualized treatment of HCC.

## Data Availability

The datasets presented in this study can be found in online repositories. The names of the repository/repositories and accession number(s) can be found in the article/Supplementary Material.
