# Supplementary material for: An m6A-Related lncRNA Signature Predicts the Prognosis of Hepatocellular Carcinoma
Source: Front Pharmacol. 2022 Mar 30;13:854851. doi: 10.3389/fphar.2022.854851 (PMC9006777; doi:10.3389/fphar.2022.854851)
Supplement: Supplementary file 2 [file DataSheet2.docx]

**Supplementary Table1.** Association between m6A-correlated lncRNA signature and clinicopathologic characteristics in GSE14520 HCC cohort

| Variables | GSE14520 (n=218) | | |
| --- | --- | --- | --- |
|  | Low risk group | High risk group | P-value |
| Age(years) |  |  | 0.069 |
| ≤60 | 86 | 93 |  |
| >60 | 25 | 14 |  |
| Sex |  |  | 0.914 |
| male | 96 | 92 |  |
| female | 15 | 15 |  |
| Cirrhosis |  |  | 0.366 |
| no | 11 | 7 |  |
| yes | 100 | 100 |  |
| TNM stage |  |  | **0.004** |
| I/II | 95 | 74 |  |
| III/IV | 16 | 33 |  |
| Survival status |  |  | 0.059 |
| alive | 75 | 59 |  |
| dead | 36 | 48 |  |

Notes: HCC: hepatocellular carcinoma; TNM: Tumor Node Metastasis.

**Supplementary Table2**. Univariate and multivariate regression analyses of OS in HCC patients of the GSE14520 dataset.

| Variable | Univariate analysis | | | Multivariate analysis | | |
| --- | --- | --- | --- | --- | --- | --- |
|  | p | Hazard ratio | 95%confidence  interval | p | Hazard ratio | 95%confidence interval |
| Age (>60 / ≤60) | 0.715 | 0.899 | 0.506-1.596 |  |  |  |
| Sex (female / male) | 0.150 | 0.586 | 0.283-1.214 |  |  |  |
| TNM stage (IV+III/II+I) | **<0.001** | **3.487** | **2.223-5.470** | **<0.001** | **3.075** | **1.937-4.882** |
| Cirrhosis (yes/no) | **0.032** | **4.650** | **1.143-18.912** | 0.054 | 3.984 | 0.977-16.248 |
| Risk score (high/low) | **0.022** | **1.660** | **1.077-2.559** | 0.173 | 1.362 | 0.874-2.124 |

Notes: TNM: Tumor Node Metastasis; OS: overall survival; HCC: hepatocellular carcinoma.

**Supplementary Table3**. significantly enriched KEGG pathways in TCGA high risk groups using GSEA (PDR<0.05)

| ID | PATHWAY | SIZE | ES | NES | NOM p-val | FDR q-val |
| --- | --- | --- | --- | --- | --- | --- |
| 1 | KEGG_BASE_EXCISION_REPAIR | 35 | 0.76 | 2.06 | 0 | 0.005 |
| 2 | KEGG_SPLICEOSOME | 127 | 0.79 | 2.05 | 0 | 0.003 |
| 3 | KEGG_PYRIMIDINE_METABOLISM | 98 | 0.62 | 2.04 | 0 | 0.002 |
| 4 | KEGG_RNA_DEGRADATION | 59 | 0.71 | 2.01 | 0 | 0.002 |
| 5 | KEGG_NUCLEOTIDE_EXCISION_REPAIR | 44 | 0.73 | 2.01 | 0 | 0.003 |
| 6 | KEGG_DNA_REPLICATION | 36 | 0.83 | 1.97 | 0 | 0.006 |
| 7 | KEGG_OOCYTE_MEIOSIS | 113 | 0.61 | 1.94 | 0 | 0.009 |
| 8 | KEGG_CELL_CYCLE | 125 | 0.69 | 1.93 | 0 | 0.009 |
| 9 | KEGG_BLADDER_CANCER | 42 | 0.64 | 1.89 | 0 | 0.018 |
| 10 | KEGG_HOMOLOGOUS_RECOMBINATION | 28 | 0.76 | 1.88 | 0.002 | 0.02 |
| 11 | KEGG_PURINE_METABOLISM | 157 | 0.53 | 1.87 | 0 | 0.022 |
| 12 | KEGG_MISMATCH_REPAIR | 23 | 0.77 | 1.85 | 0.002 | 0.023 |

Notes: KEGG: Kyoto Encyclopedia of Genes and Genomes; TCGA: The Cancer Genome Atlas; GSEA: gene set enrichment analysis; ES: Enrichment score; NES: Normalized enrichment score.

**Supplementary Table4**. significantly enriched KEGG pathways in TCGA low risk groups using GSEA (PDR<0.05)

| ID | PATHWAY | SIZE | ES | NES | NOM p-val | FDR q-val |
| --- | --- | --- | --- | --- | --- | --- |
| 1 | KEGG_COMPLEMENT_AND_COAGULATION_CASCADES | 69 | -0.84 | -2.38 | 0 | 0 |
| 2 | KEGG_DRUG_METABOLISM_CYTOCHROME_P450 | 71 | -0.75 | -2.22 | 0 | 0 |
| 3 | KEGG_RETINOL_METABOLISM | 64 | -0.75 | -2.19 | 0 | 0 |
| 4 | KEGG_PRIMARY_BILE_ACID_BIOSYNTHESIS | 16 | -0.94 | -2.07 | 0 | 0 |
| 5 | KEGG_FATTY_ACID_METABOLISM | 42 | -0.84 | -2.02 | 0 | 0.001 |
| 6 | KEGG_VALINE_LEUCINE_AND_ISOLEUCINE_DEGRADATION | 43 | -0.84 | -2.01 | 0 | 0.001 |
| 7 | KEGG_TRYPTOPHAN_METABOLISM | 39 | -0.72 | -1.99 | 0 | 0.001 |
| 8 | KEGG_METABOLISM_OF_XENOBIOTICS_BY_CYTOCHROME_P450 | 69 | -0.65 | -1.96 | 0 | 0.001 |
| 9 | KEGG_GLYCINE_SERINE_AND_THREONINE_METABOLISM | 31 | -0.74 | -1.91 | 0.006 | 0.004 |
| 10 | KEGG_BUTANOATE_METABOLISM | 34 | -0.7 | -1.87 | 0.004 | 0.006 |
| 11 | KEGG_STEROID_HORMONE_BIOSYNTHESIS | 55 | -0.61 | -1.83 | 0.004 | 0.009 |
| 12 | KEGG_PROPANOATE_METABOLISM | 32 | -0.72 | -1.82 | 0.008 | 0.01 |
| 13 | KEGG_PEROXISOME | 78 | -0.6 | -1.76 | 0.015 | 0.017 |
| 14 | KEGG_ASCORBATE_AND_ALDARATE_METABOLISM | 25 | -0.7 | -1.76 | 0.006 | 0.017 |
| 15 | KEGG_PPAR_SIGNALING_PATHWAY | 69 | -0.56 | -1.7 | 0.008 | 0.027 |
| 16 | KEGG_BETA_ALANINE_METABOLISM | 22 | -0.67 | -1.7 | 0.017 | 0.026 |
| 17 | KEGG_ALANINE_ASPARTATE_AND_GLUTAMATE_METABOLISM | 30 | -0.53 | -1.64 | 0.015 | 0.046 |

Notes: KEGG: Kyoto Encyclopedia of Genes and Genomes; TCGA: The Cancer Genome Atlas; GSEA: gene set enrichment analysis; ES: Enrichment score; NES: Normalized enrichment score.


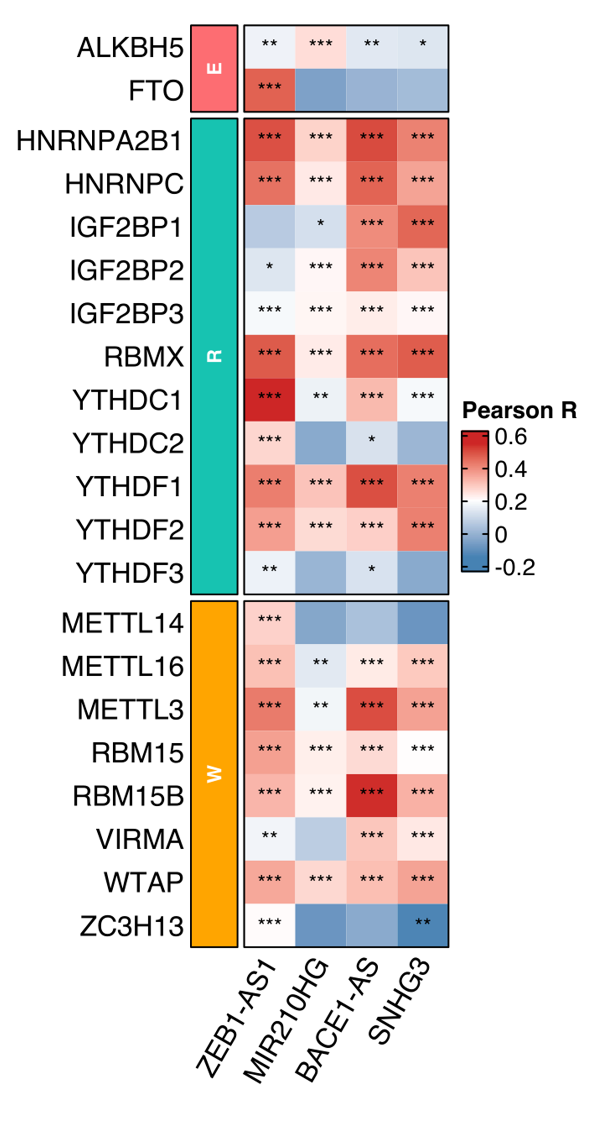


**Supplementary Figure1**. The heatmap of correlations between the four screened m6A-related lncRNAs and all m6A regulators


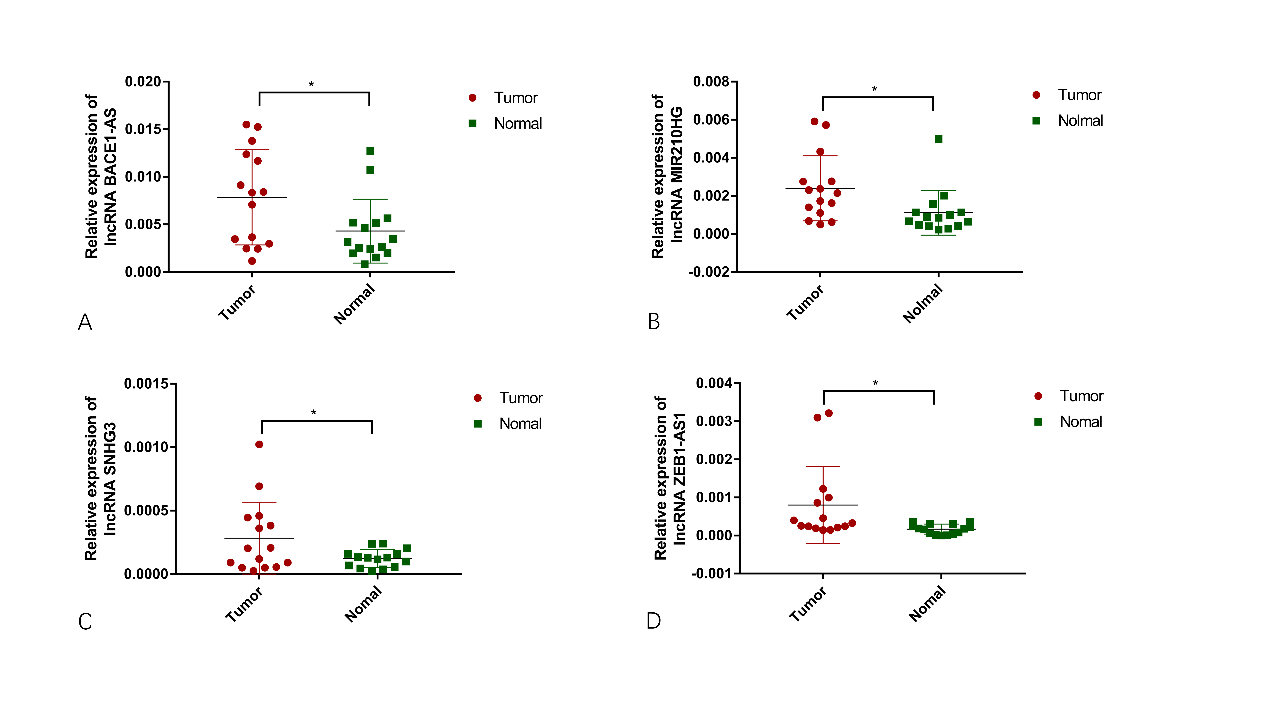


**Supplementary Figure2**. Relative mRNA expression of the four selected LncRNAs.

Note:(A) BACE1-AS mRNA expression between Tumor and normal tissues; (B) MIR210HG mRNA expression between Tumor and normal tissues; (C) SNHG3 mRNA expression between Tumor and normal tissues; (D) ZEB1-AS1 mRNA expression between Tumor and normal tissues.


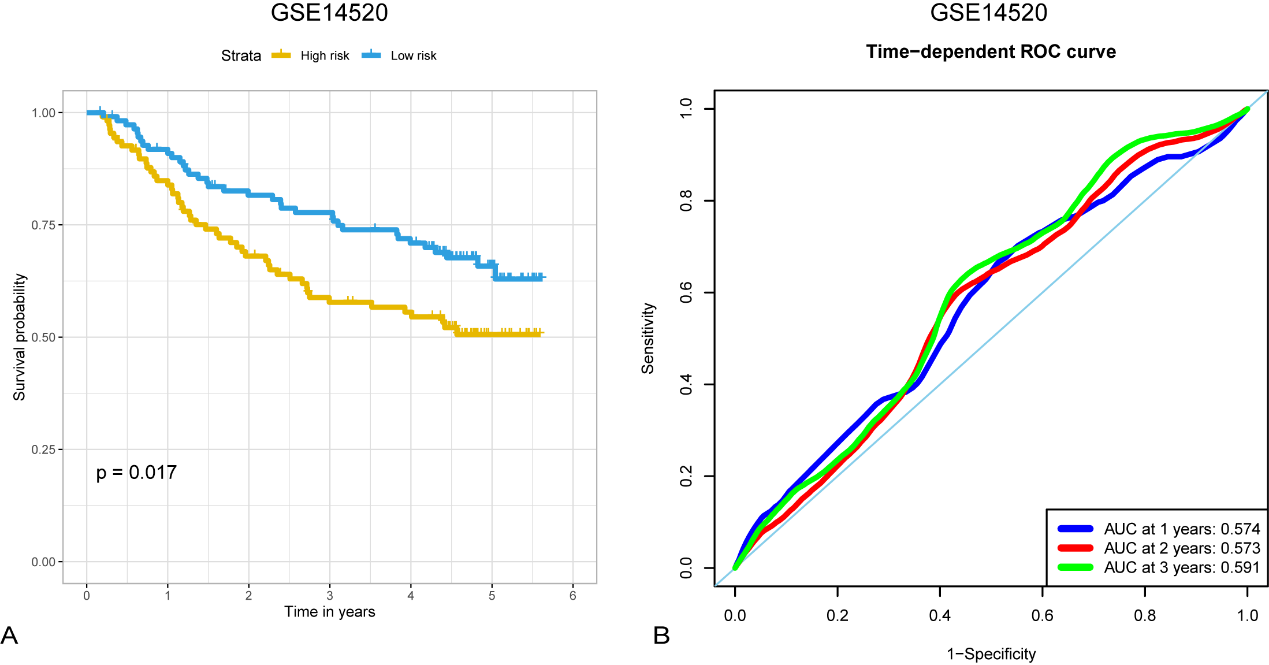


**Supplementary Figure3.** The predictive value of m6A-related lncRNA signature in the GSE14520 dataset.

(A). The survival curve of HCC patients in the high and low-risk groups in the GSE14520 dataset; (B). The ability of m6A-related lncRNA signature to predict the overall survival of HCC patients in the GSE14520 dataset.


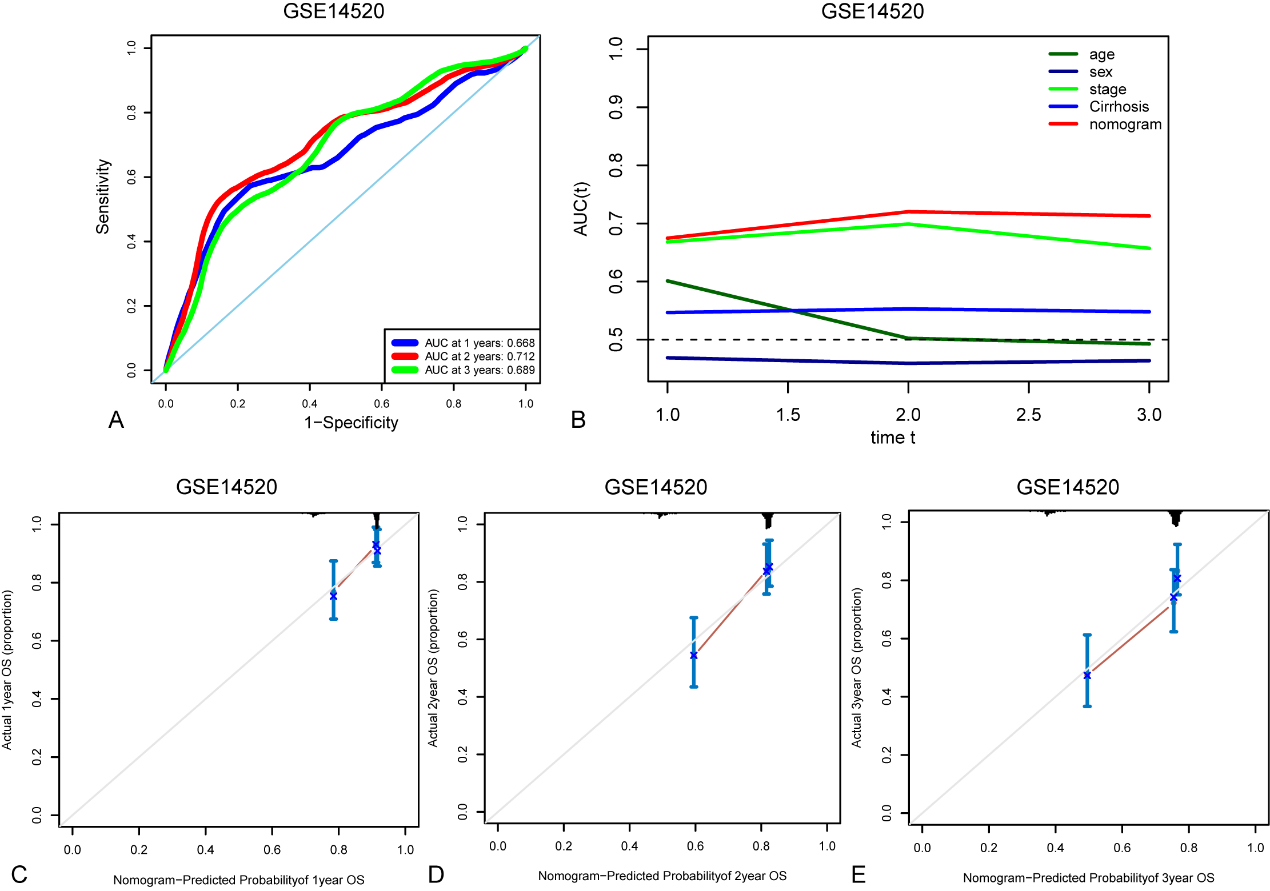


**Supplementary Figure4**. Nomogram external validation using GSE14520 dataset.

(A)The predictive value of the nomogram for the 1-year, 2-year, and 3-year OS rates of HCC patients in GSE14520 dataset; (B) Comparison of the predictive value of nomogram and other clinicopathological features in GSE14520 dataset; (C-E) 1-year, 2-year, and 3-year calibration curve of OS-related nomogram in the GSE14520 dataset.
